# Supplementary material for: Highly efficient non-relativistic Edelstein effect in nodal p-wave magnets
Source: Nat Commun. 2025 Aug 7;16:7270. doi: 10.1038/s41467-025-62516-0 (PMC12332020; doi:10.1038/s41467-025-62516-0)
Supplement: Supplementary file 1 — Supplementary Information [file 41467_2025_62516_MOESM1_ESM.pdf]

Supplementary Information

Highly Efficient Non-relativistic Edelstein effect in nodal p-wave magnets

Atasi Chakraborty[1*], Anna Birk Hellenes[1], Rodrigo Jaeschke-Ubiergo[1], Tomás Jungwirth[2,3], Libor Šmejkal[4,5,1,2 *], and Jairo Sinova[1,6*]

[1]*Institut für Physik, Johannes Gutenberg Universität Mainz, D-55099 Mainz, Germany*

[2]*Institute of Physics, Academy of Sciences of the Czech Republic, Cukrovarnická 10, 162 00 Praha 6, Czech Republic*

[3]*School of Physics and Astronomy, University of Nottingham, NG7 2RD, Nottingham, United Kingdom*

[4]*Max Plank Institute for the Physics of Complex Systems, Nöthnitzer Str. 38, 01187 Dresden, Germany*

[5]*Max Planck Institute for Chemical Physics of Solids, Nöthnitzer Str. 40, 01187 Dresden, Germany*

[6]*Department of Physics, Texas A & M University, College Station, Texas 77843-4242, USA*

[*]*atasi.chakraborty@uni-mainz.de, lsmejkal@pks.mpg.de, sinova@uni-mainz.de*

August 1, 2025

Contents

1 Analytical derivation of spin isotropic bands **3**

2 Isotropic spin-split bands **4**

3 Derivation of the minimal model **5**

4 Coplanar $\mathcal{T}$t− spin order of bi-kagome magnet with spin canting $\theta_s = 0^\circ$ **7**

5 Effect of spin-orbit coupling on Edelstein effect of CeNiAsO **8**

6 Scattering time independent comparison of response for CeNiAsO and $LuFeO_3$ **10**

7 Effect of electronic correlation **11**

8 Complete list of coplanar p-wave magnets and relevant non-relativistic non-equilibrium spin susceptibility components **13**

1 Analytical derivation of spin isotropic bands

The intra-band non-equilibrium spin density can be expressed as

$$\delta\mathbf{s}_{\text{intra}} = \frac{e\hbar}{2\Gamma}\int \frac{d^2k}{(2\pi)^2}\sum_{\alpha}\mathbf{S}_{\mathbf{k}\alpha}(\mathbf{E}\cdot\mathbf{v})_{\mathbf{k}\alpha}\delta(E_{\mathbf{k}\alpha}-E_F) \tag{1}$$

For one band with isotropic spin i.e. spin density is independent of momentum as shown in Fig. 1(a), we can take the S_k out of the summation. Considering, electric field $\mathbf{E} \mid\mid \mathbf{x}$, we can rewrite the expression of Fig. 1(a) for each red and blue band as

$$\delta\mathbf{s}_{\text{intra},\alpha} = \frac{e\hbar \mathrm{E} S}{2\Gamma}\int \frac{d^2k}{(2\pi)^2} v_{x,\mathbf{k},\alpha}\ \delta(E_{\mathbf{k}\alpha}-E_F)\ . \tag{2}$$

Below we analytically derive the Edelstein effect for one band. The band energy can be expressed with the following expression

$$\begin{aligned} E_{\mathbf{k}} &= \sqrt{k_x^2+k_y^2} = |\mathbf{k}|; & (3)\\ v_x &= \frac{\partial E_{\mathbf{k}}}{\partial k_x} = \frac{k_x}{\sqrt{k_x^2+k_y^2}} = \frac{k\cos\theta}{|k|} & (4) \end{aligned}$$

Hence, the eqn 2 can be rewritten for this energy dispersion as:

$$\begin{aligned} \delta\mathbf{s}_{\text{intra}} &= \frac{e\hbar \mathrm{E} S}{2\Gamma}\int_k\int_\theta k dk d\theta \frac{k\cos\theta}{|k|}\delta(E_{\mathbf{k}}-E_F) \\ &= \frac{e\hbar \mathrm{E} S}{2\Gamma}\int_{\theta=0}^{2\pi}\cos\theta d\theta \int_{E_{\mathbf{k}}} E_{\mathbf{k}}\delta(E_{\mathbf{k}}-E_F)dE_{\mathbf{k}} \\ &= 0 \end{aligned} \tag{5}$$

For an isotropic spin band, the non-equilibrium spin accumulation vanishes for individual bands. It can be understood from a very simple argument: as the band has constant spin expectation at each point, the effect of the electric field only shifts the Fermi surface. The spins acquired along $\mathbf{E}$ are compensated by the empty region of the right, as can be seen by following each band movement. Hence no net non-equilibrium spin accumulation. This is true even is the centre of the circle if shifted from the origin. The energy expression of red and blue bands are $E_{\mathbf{k}} = |\mathbf{k}\pm\mathbf{k_0}|$ respectively. As long as the slope of left and right (do not get confused with $\pm k$) of each band is the same, the

individual contribution $\delta s_{\text{intra}} = 0$.

2 Isotropic spin-split bands

In this section, we establish the importance of spin-polarization modulation of p-wave magnets originating from exchange-dependent hopping for the non-equilibrium spin-accumulation. For comparison we choose two scenarios: 1. circular isotropic spin bands oppositely shifted in the opposite momentum 2. Deformed isotropic bands preserving time-reversal symmetry in momentum space utilizing the following energy dispersion,

$$E_{\mathbf{k}}^{\pm} = t[(k_x \pm k_0)^2 + k_y^2(1 \mp a k_x)]; \tag{6}$$

Here, $\pm$ represents the up and down spin channels, respectively. We set, $t = 1eV, k_0 = 0.6\text{Å}^{-1}$ and $a = 0\text{Å}$ for Fig. 1 (a). Our theoretical calculations suggest that not only the total NREE but also the NREE vanishes for the individual spin channel, as expected from our analytical calculation of SI section 1. The finite value of a distorts each spin channel but in the opposite momentum direction. We plot the constant energy surface for $E_{\mathbf{k}} = 3.0t$ with $a = 0.4\text{Å}$ in Fig. 1 (b). The fixed energy contour has a dispersion very similar to the p-wave minimal model of the main manuscript. However, in this case, the bands do not contain any polarization modulation of spin, which is characteristic of an odd-parity p-wave electronic structure. The NREE for this distorted disentangled spin band vanishes at each energy in a similar way to the example of the shifted spin circles of Fig. 1 (a). Therefore, the modulation of spin-polarization, combined with the distortion, is essential to obtain the finite charge-spin conversion.

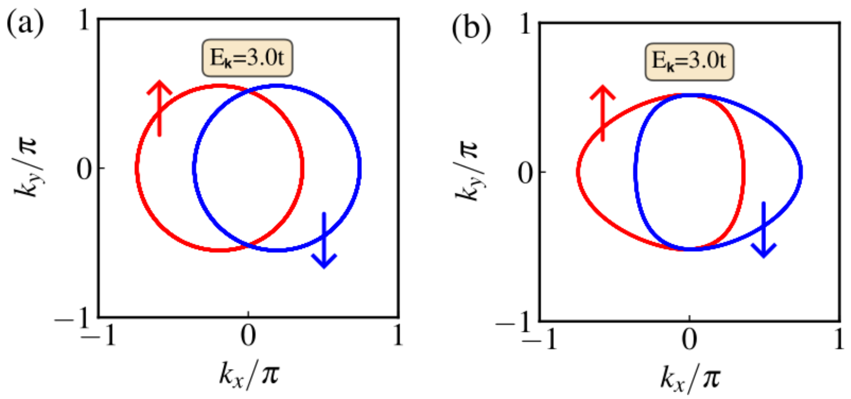

Supplementary Figure 1. (a) The disentangled isotropic spin circles shifted oppositely in k_x direction and preserved time-reversal symmetry (TRS) in momentum space. (b) Distorted isotropic spin circles for finite a. The opposite distortion of spin bands preserves TRS in momentum space.

3 Derivation of the minimal model

In this section, we include a detailed derivation of the minimal Hamiltonian model from lattice geometry. In Fig. 2 we show the square lattice geometry with two sublattices A and B (in purple and pink color circles) and corresponding exchange-driven hopping paths through the arrow inside the circle for each nearest neighbor path. The exchange dependent hopping terms include σ_x (σ_y) along nearest neighbor connecting through $\frac{\delta \mathbf{x}}{2}$ ($\delta \mathbf{y}$). The direction of the arrow represents the sign of each of the σ components. The total Hamiltonian containing nearest neighbor interactions can be expressed in the following form:

$$H = \sum_{\langle ij \rangle} t c_i^\dagger c_j + t_J \left[\sum_{\langle ij \rangle, x^+} \sigma_x c_B^\dagger c_A - \sum_{\langle ij \rangle, x^-} \sigma_x c_B^\dagger c_A - \sum_{\langle ij \rangle, x^+} \sigma_x c_A^\dagger c_B + \sum_{\langle ij \rangle, x^-} \sigma_x c_A^\dagger c_B + \sum_{\langle ij \rangle} \sigma_y c_A^\dagger c_A - \sum_{\langle ij \rangle} \sigma_y c_B^\dagger c_B \right] \tag{7}$$

The first term is the spin-independent nearest neighbor square lattice tight-binding Hamiltonian with dispersion $2t(\cos\frac{k_x \delta_x}{2}\tau_1 + \cos k_y \delta_y)$. In the second term, the $x^+(y^+)$ and $x^-(y^-)$ represent nearest neighbor hoppings in the positive and negative x (y) axes. Here, $c^\dagger(c) = \frac{1}{\sqrt{V}} \sum_{\mathbf{k}} e^{+(-)i\mathbf{k}\cdot\mathbf{r}}\, c_k^\dagger (c_k)$ represents the creation (annihilation) operator. The individual sublattice-dependent interaction

terms can be expressed as,

$$\begin{aligned} H_{AA} \quad &: \quad t_J\sigma_y(e^{ik_y\delta_y}+e^{-ik_y\delta_y}) \;= 2\sigma_y\text{cos}k_y\delta_y \\ H_{BB} \quad &: \quad -t_J\sigma_y(e^{ik_y\delta_y}+e^{-ik_y\delta_y}) \;= -2\sigma_y\text{cos}k_y\delta_y \\ H_{AB} \quad &: \quad t_J\sigma_x(-e^{\frac{ik_x\delta_x}{2}}+e^{\frac{-ik_x\delta_x}{2}}) \;= -2i\sigma_x\text{sin}\frac{k_x\delta_x}{2} \\ H_{BA} \quad &: \quad t_J\sigma_x(e^{\frac{ik_x\delta_x}{2}}-e^{\frac{-ik_x\delta_x}{2}}) \;= 2i\sigma_x\text{sin}\frac{k_x\delta_x}{2} \end{aligned} \tag{8}$$

Hence the complete Hamiltonian can be expressed in a compact form as,

$$H = 2t(\text{cos}\frac{k_x\delta_x}{2}\tau_1+\text{cos}k_y\delta_y)+2t_J(\sigma_x\text{sin}\frac{k_x\delta_x}{2}\tau_2+\sigma_y\text{cos}k_y\delta_y\tau_3)\ . \tag{9}$$

Here, Pauli matrices τ are defined in the A and B sublattice space, and Pauli matrices σ are defined in the spin space.

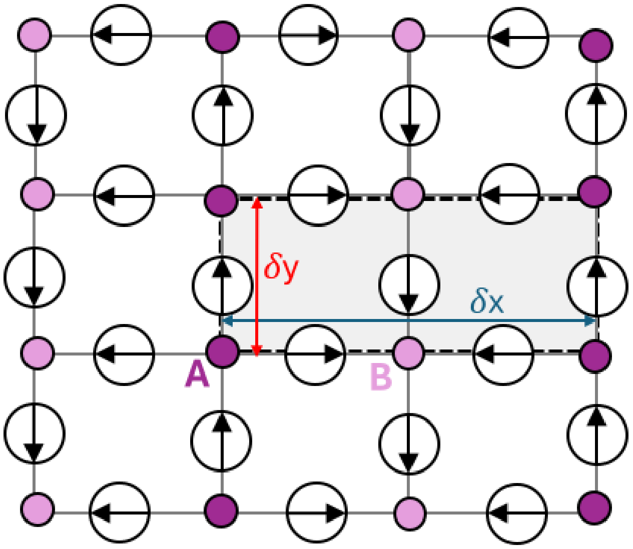

Supplementary Figure 2. Purple and pink colored circles represent the A and B sublattices within the square lattice geometry. Different exchange dependent hopping paths are shown with arrowed circles. The direction of the arrow signifies the σ matrix components involved with the hopping. The unit cell is marked in the grey shaded box.

4 Coplanar $\mathcal{T}\mathbf{t}-$ spin order of bi-kagome magnet with spin canting $\theta_s = 0°$

In this section, we explore the characteristics of coplanar spin arrangement other than the 120° arrangement as discussed in section 5 of the main manuscript. Here, we consider a spin arrangement where two of the spins are along $\hat{x}$, and one is along $\hat{y}$ within an individual triangle (see Fig. 3a), breaking the overall inversion of the lattice. The two consecutive red and blue triangles are still connected by the $\mathcal{T}\mathbf{t}$ symmetry. By our construction, this hypothetical spin arrangement also satisfies the criteria for the p-wave texture. We find similar to the 120° spin arrangement; only the out-of-plane spin component survives in the momentum space due to the presence of the $[C_{2\perp}||\,\mathbf{t}\,]$ symmetry. We have plotted the χ_S^{zx} and χ_S^{zy} components of the susceptibility tensor in Fig. 3 (c). We find the order of magnitude is similar to that of 120° spin arrangement. We plot the angular distribution of the χ for three different energy cuts $E_{\mathbf{k}} = -0.22$ eV, -1.0 eV and $+0.2$ eV in Fig. 3 (d), (e) and (f) respectively. Interestingly, we find the anisotropy axis of the dumbell changes with energy. However, the anisotropy axis of χ_S was fixed to $\mathbf{E}||(-\frac{\sqrt{3}}{2}\hat{x} + \frac{1}{2}\hat{y})$ independent of the chemical potential for the 120° spin arrangement of the main manuscript.

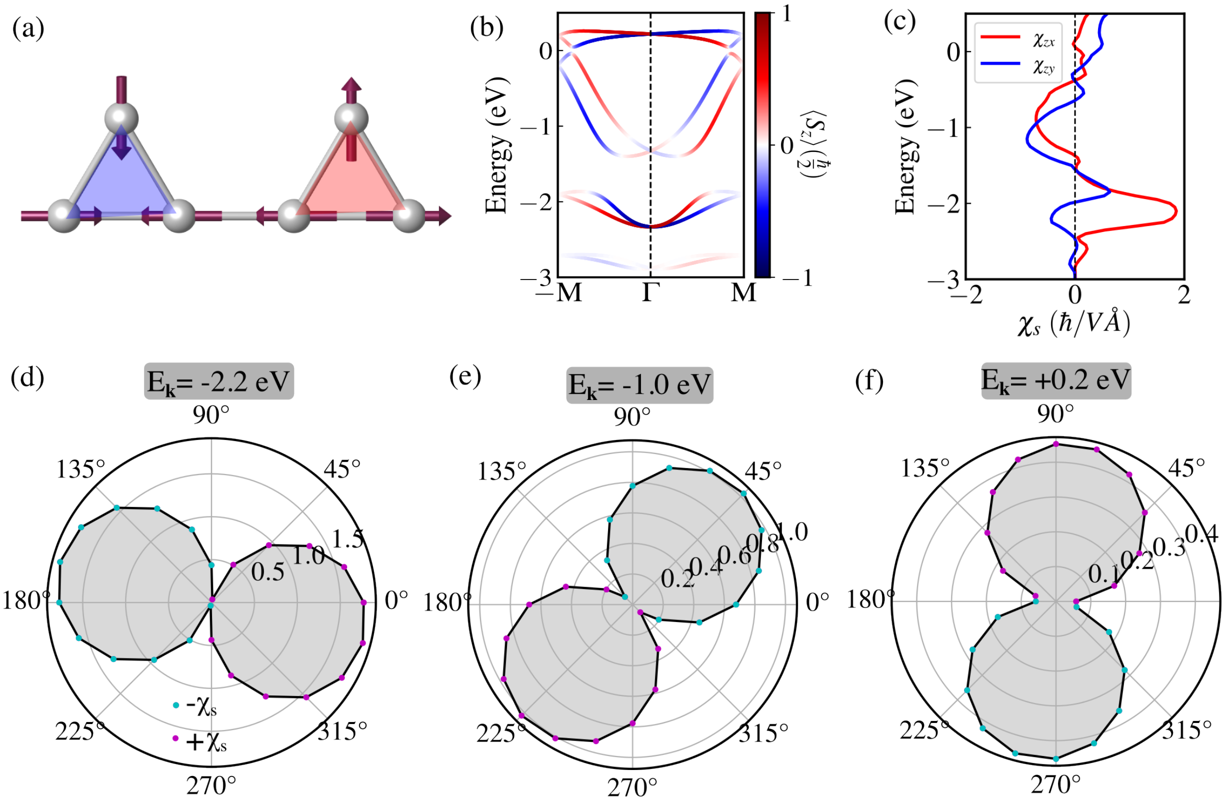

Supplementary Figure 3. (a) Direct space coplanar noncollinear magnetic order of the bi-kagome lattice. The magnetic order exhibits combined time-reversal and lattice translation symmetry $\mathcal{T}\mathbf{t}$. (b) Calculated nonrelativistic odd-parity wave spin-split band structure along the $-M\Gamma M$ path with out-of-plane spin projection S_z.(c) The variation of zx and zy components of non-equilibrium intra-band susceptibility density with energy. The angular distribution of χ_S (in units of $\hbar/V$Å) for energy -2.2 eV (d), -1.0 eV (e) and +0.2 eV (f) w.r.t change in the electric field direction. The cyan and magenta circles represent the negative and positive values of χ_S.

5 Effect of spin-orbit coupling on Edelstein effect of CeNiAsO

In this section, we investigate how relativistic effects modify the NREE signal of CeNiAsO. Given that Ce, the magnetic building block, is a rare earth element, its heavy nuclei generate strong electric fields, leading to a considerable relativistic correction. In the presence of SOC, the symmetries adhere to the relevant magnetic point group operations. The generators of the ground state magnetic point group $21'$ are $\{E, C_{2y}\mathcal{T}, C_{2y}\}$. In the presence of SOC, the $[C_{2\perp}||\,\mathbf{t}\,]$ is no longer a symmetry operation of the magnetic space group. Therefore, spin-polarization can allow the in-plane component to be combined with the out-of-plane polarization texture. We have plotted individual spin-component resolved band dispersion along high-symmetry $X\Gamma X$ direction in Fig. 4 (a-c) and $M\Gamma M$ direction in Fig. 4 (d-f). The spin components connect anti-symmetrically oppo-

site momentum points. The susceptibility tensor in the relativistic regime allowed by the magnetic point group symmetry has the following form:

$$\begin{bmatrix} \chi_{xx} & 0 & \chi_{xz} \\ 0 & \chi_{yy} & 0 \\ \chi_{zx} & 0 & \chi_{zz} \end{bmatrix}$$

We find SOC triggers the finite contribution of χ_{xx}, χ_{xz} and χ_{yy} which originates completely due to relativistic origin. In Fig. 4. (g) and (h) we plot the diagonal and off-diagonal components of the susceptibility tensor for the non-equilibrium spin accumulation density near the Fermi energy. The susceptibility components originated due to SOC are marked with olive arrows in Fig. 4. Our calculation suggests we get finite contribution only for those components of χ_S, which are allowed by symmetry.

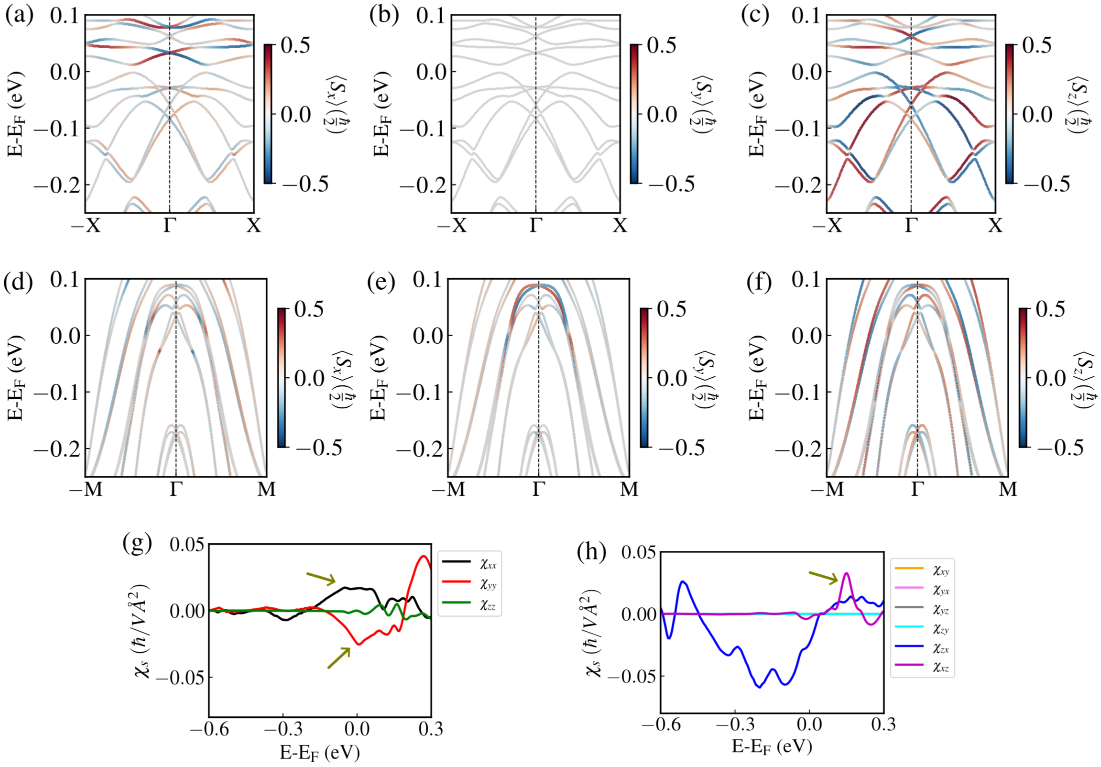

Supplementary Figure 4. The top and middle panels show the relativistic band dispersion along high symmetry $X\Gamma X$ and $M\Gamma M$ paths, respectively. The S_x, S_y and S_z spin components are shown with color maps in (a,d), (b,e) and (c,f). The diagonal and off-diagonal components of non-equilibrium spin accumulation density are shown in panels (g) and (h), respectively. The olive arrow marks responses coming solely due to relativistic origin.

6 Scattering time independent comparison of response for CeNiAsO and $LuFeO_3$

To compare the NREE of the p-wave candidate CeNiAsO and The non-equilibrium spin accumulation scales with the scattering time (τ), its exact quantitative value can vary somewhat depending on the system. The typical values of scattering time are estimated using the following formula [1]

$$\tau = \left(\frac{0.22}{\rho}\right)\left(\frac{r_s}{a_0}\right)^3 \times 10^{-14} \text{ sec} . \tag{10}$$

ρ, r_s, and a_0 represent resistivity (in micro-ohm cm), the radius of a sphere with a volume equal to the volume per conduction electron and Bohr radius, respectively. At room temperature, τ typically falls within the range of 10^{-14} to 10^{-15} seconds. However, an accurate estimation of τ for any given system requires theoretical modelling of the experimental data, which is currently unavailable for both systems of interest.

Below, we define the τ independent quantity as ratio of $\mathcal{T}$-even Edelstein susceptibility (χ) with the longitudinal ohmic conductivity (σ_D). In the top panel, we have plotted the Ohmic conductivity for CeNiAsO (Fig. 5 (a)) and $LuFeO_3$ (Fig. 5 (b)) around the Fermi energy (E_F) by using the following expression,

$$\sigma_{ij} = \frac{2\pi}{\Gamma}\left(\frac{e^2}{h}\right)\int [d\mathbf{k}] \sum_n \frac{\delta E_{\mathbf{k}n}}{\delta k_i}\frac{\delta E_{\mathbf{k}n}}{\delta k_j}\left(-\frac{\delta f_0}{\delta E_{\mathbf{k}}}\right)_{E_{\mathbf{k}}=E_n} . \tag{11}$$

We set Γ =0.01 eV, the identical value we choose to calculate the Edelstein response. Interestingly, we see LuFeO3 shows isotropic in-plane Drude conductivity, whereas, in CeNiAsO, the unconventional p-wave magnetic phase spontaneously breaks the crystal symmetry, resulting in a large resistive anisotropic longitudinal response. In panels Fig. 5 (c) and ((d), we have plotted the ratio of the highest component of $\mathcal{T}$-even Edelstein response with σ_D, to make it a scattering time independent parameter. We have also included the zoomed-in view in the inset in the energy region where the σ_D is highest and expected to show minimum χ/σ_D values for CeNiAsO. We find that the minimum value of the ratio for *p*-wave CeNiAsO surpasses more than 10 times that of $LuFeO_3$.

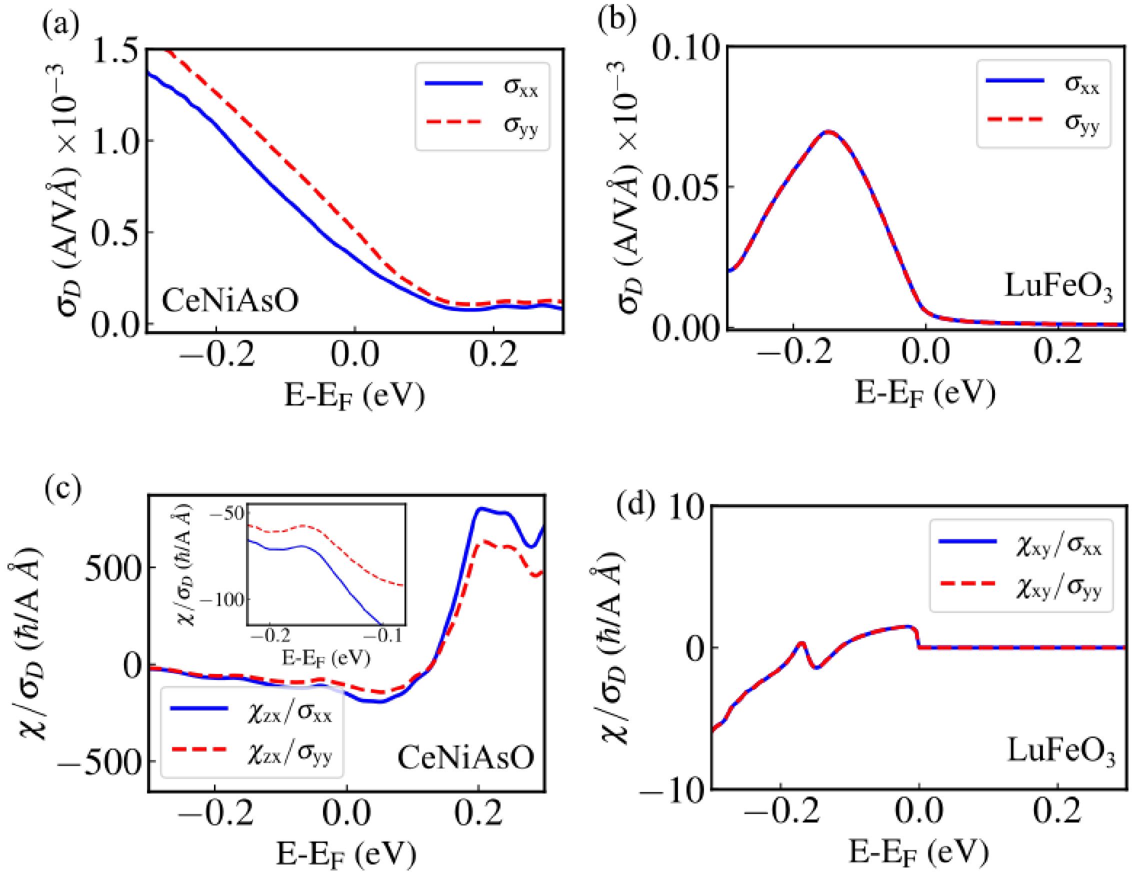

Supplementary Figure 5. Longitudinal conductivities calculated along the x and y axis for (a) CeNiAsO and (b) LuFeO3. The scattering time-independent parameter (χ/σ_D) for (c) CeNiAsO and (d) LuFeO3. We have plotted the highest global Edelstein response tensor component to calculate the ratio, which is χ_{zx} for CeNiASO and χ_{xy} for LuFeO3, respectively.

7 Effect of electronic correlation

In this section, we analyse the evolution of moment at Ce atom with Coulomb correlation. As a lanthanide element, cerium typically exhibits strong electron correlations at its atomic sites, which are essential for maintaining localized f-orbitals. The typical choice of Hubbard U for Ce atoms lie within the range of 4.3-6.7 eV [2]. In our calculations, we used λ = 6 eV/μ_B^2, constraining non-collinear magnetic moments for different choices of Hubbard U at Ce sites within the GGA+U scheme. In RFig. 2a, we have plotted the orbital projected band-dispersion of the Ce-f states along high-symmetry paths with U =0 eV. We find that the calculation without Hubbard interaction can successfully capture the strongly localized nature of the Ce-f bands near the Fermi energy. Interestingly, the magnetic moment at the Ce atom i.e. (±0.35, ±0.26, 0) μ_B within U =0 eV calculations

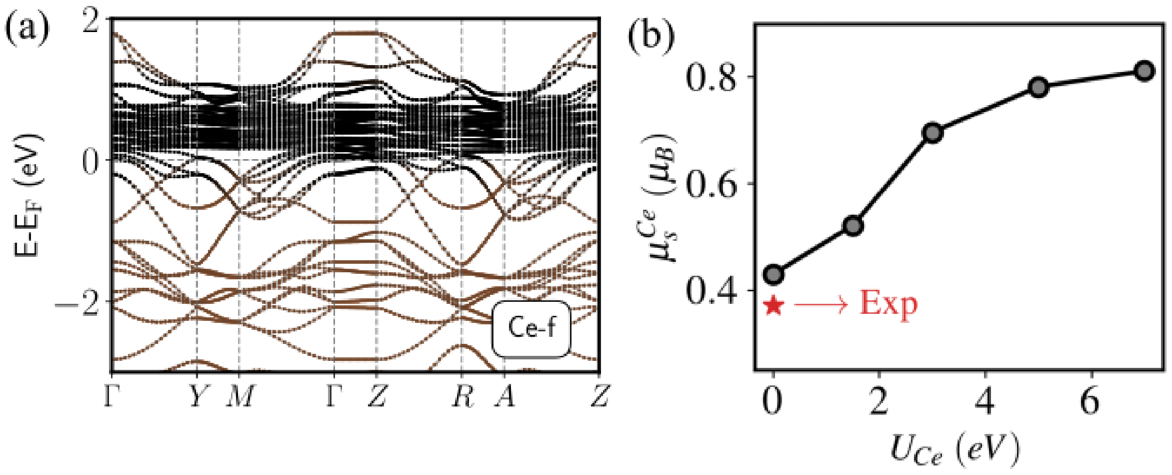

Supplementary Figure 6. (a) The orbital decomposed band dispersion of Ce-f states (black) on the total energy dispersion. (b) Variation of magnetic moment at Ce site for different values of Hubbard U. The experimentally observed moment in μ-SR experiment is shown in [4] is marked with a red star.

agrees well with the experimentally obtained effective moment value, $\mu_{eff} = (\pm 0.3, \pm 0.22, 0)\mu_B$, below 7.6 K. The contribution of localized Ce-f states is closer to the conduction band edge (see RFig. 2a) as similar to the other high-temperature phases of CeNiAsO reported in the literature [3].

Our further theoretical calculations suggest, as per the expectation, that the increase of Hubbard U at the magnetic site enhanced the magnetic moment at Ce sites, as shown in RFig. 2b. However, the increased moment for finite values of Hubbard U deviates the theoretical moment vastly from the experimental effective moment (marked in red star). Therefore, we have carried out our further calculations of electronic structure and responses with U =0 eV.

8 Complete list of coplanar p-wave magnets and relevant non-relativistic non-equilibrium spin susceptibility components

Supplementary Table 1. Spin symmetry constrained NREE tensor in co-planar p-wave magnetic material candidates. The third column represents the axis of spin-polarization. Those spin-polarization axes, which are not aligned to either of the crystal axes a, b or c, are expressed with precision up to a second decimal place with respect to the crystal axes. The generators of the non-trivial spin poing group are included in the fourth column. All the symmetries are expressed in the lattice coordinates provided in the MAGNDATA database [5]. The fifth column shows the symmetry-enforced structure of the NREE susceptibility tensor expressed in the cartesian coordinate system. In entry no. 13, for $NiPS_3$, ϵ is a negligibly small number.

No.	Name	Spin pol.	SPG generators	NREE tensor
1	CeNiAsO	[0,0,1]	$[C_{2c}\|\|E]$, $[E\|\|\mathcal{M}_b]$, $[C_{2a}\|\|C_{2b}]$	$\begin{bmatrix} 0 & 0 & 0 \\ 0 & 0 & 0 \\ \chi_{zx} & 0 & \chi_{zz} \end{bmatrix}$
2	$BiMn_2O_5$	[0,0,1]	$[C_{2b}\|\|\mathcal{M}_a]$, $[C_{2c}\|\|\mathcal{M}_c]$, $[C_{2c}\|\|E]$	$\begin{bmatrix} 0 & 0 & 0 \\ 0 & 0 & 0 \\ \chi_{zx} & 0 & 0 \end{bmatrix}$
3	Lu_2CoMnO_6	[0,1,0]	$[C_{2b}\|\|C_{2b}]$, $[C_{2b}\|\|E]$	$\begin{bmatrix} 0 & 0 & 0 \\ 0 & \chi_{yy} & 0 \\ 0 & 0 & 0 \end{bmatrix}$
4	$CsFe(MoO_4)_2$	[0,0,1]	$[C_{\bar{3}c}\|\|E]$, $[C_{2c}\|\|E]$, $[C_{2d}\|\|iC_{\bar{3}c}]$; $d = (210)$	$\begin{bmatrix} 0 & 0 & 0 \\ 0 & 0 & 0 \\ 0 & 0 & \chi_{zz} \end{bmatrix}$
5	$HoMn_2O_5$	[0,0,1]	$[E\|\|\mathcal{M}_c]$, $[C_{2c}\|\|E]$	$\begin{bmatrix} 0 & 0 & 0 \\ 0 & 0 & 0 \\ \chi_{zx} & \chi_{zy} & 0 \end{bmatrix}$
6	TbC_2	[1,0,0]	$[C_{2c}\|\|P]$, $[E\|\|\mathcal{M}_c]$, $[C_{2a}\|\|E]$, $[E\|\|C_{2a}]$	$\begin{bmatrix} \chi_{xx} & 0 & 0 \\ 0 & 0 & 0 \\ 0 & 0 & 0 \end{bmatrix}$
7	$LuNiO_3$	[0,1,0]	$[C_{2b}\|\|E]$, $[E\|\|C_{2b}]$	$\begin{bmatrix} 0 & 0 & 0 \\ 0 & \chi_{yy} & 0 \\ 0 & 0 & 0 \end{bmatrix}$
8	Gd_2BaCuO_5	[0,1,0]	$[C_{2a}\|\|C_{2a}]$, $[C_{2b}\|\|E]$, $[E\|\|\mathcal{M}_b]$	$\begin{bmatrix} 0 & 0 & 0 \\ 0 & 0 & \chi_{yz} \\ 0 & 0 & 0 \end{bmatrix}$
9	$DyMn_2O_5$	[0,0,1]	$[E\|\|\mathcal{M}_z]$, $[C_{2z}\|\|E]$, $[C_{2x}\|\|\mathcal{M}_x]$	$\begin{bmatrix} 0 & 0 & 0 \\ 0 & 0 & 0 \\ \chi_{zx} & 0 & 0 \end{bmatrix}$
10	$Ca_2Cr_2O_5$	[100]	$[C_{2a}\|\|E]$, $[E\|\|\mathcal{M}_b]$, $[E\|\|C_{2a}]$	$\begin{bmatrix} \chi_{xx} & 0 & 0 \\ 0 & 0 & 0 \\ 0 & 0 & 0 \end{bmatrix}$
11	$HoNiO_3$	[0,1,0]	$[C_{2b}\|\|E]$, $[E\|\|C_{2b}]$	$\begin{bmatrix} 0 & 0 & 0 \\ 0 & \chi_{yy} & 0 \\ 0 & 0 & 0 \end{bmatrix}$
12	$Yb_2Cu_2O_5$	[0,0,1]	$[C_{2b}\|\|\mathcal{M}_a]$, $[C_{2c}\|\|E]$	$\begin{bmatrix} 0 & 0 & 0 \\ 0 & 0 & 0 \\ \chi_{zx} & 0 & 0 \end{bmatrix}$

No.	Name	Spin pol.	SPG generators	NREE tensor
13	$NiPS_3$	n=[-0.49,0.25,-0.83]	$[C_{2n}\|\|E],\ [E\|\|C_{2b}]$	$\begin{bmatrix} 0 & \chi_{xy} & 0 \\ 0 & -2\chi_{xy} & 0 \\ 0 & (-4+\epsilon)\chi_{xy} & 0 \end{bmatrix}$
14	$DyMn_2O_5$	[0,0,1]	$[E\|\|\mathcal{M}_c],\ [C_{2c}\|\|E]$	$\begin{bmatrix} 0 & 0 & 0 \\ 0 & 0 & 0 \\ \chi_{zx} & \chi_{zy} & 0 \end{bmatrix}$
15	$La_{\frac{1}{3}}Ca_{\frac{2}{3}}MnO_3$	[0,1,0]	$[C_{2c}\|\|C_{2a}],\ [C_{2b}\|\|\mathcal{M}_b],\ [C_{2b}\|\|E]$	$\begin{bmatrix} 0 & 0 & 0 \\ 0 & 0 & \chi_{yz} \\ 0 & 0 & 0 \end{bmatrix}$
16	$La_{\frac{1}{3}}Ca_{\frac{2}{3}}MnO_3$	[0,0,1]	$[C_{2a}\|\|C_{2a}],\ [C_{2c}\|\|\mathcal{M}_c],\ [C_{2c}\|\|E]$	$\begin{bmatrix} 0 & 0 & 0 \\ 0 & 0 & 0 \\ 0 & 0 & \chi_{zz} \end{bmatrix}$
17	$Na_2Co_2TeO_2$	n=[1,1,0]	$[E\|\|C_{2n}],\ [C_{2n}\|\|E],\ [C_{2c}\|\|C_{2c}]$	$\begin{bmatrix} \chi_{xx} & \frac{1}{\sqrt{3}}\chi_{xx} & 0 \\ \frac{1}{\sqrt{3}}\chi_{xx} & \frac{1}{3}\chi_{xx} & 0 \\ 0 & 0 & 0 \end{bmatrix}$
18	$CoNb_2O_6$	[0,1,0]	$[C_{2c}\|\|C_{2a}],\ [C_{2b}\|\|E],\ [C_{2c}\|\|C_{2c}]$	$\begin{bmatrix} 0 & 0 & 0 \\ 0 & \chi_{yy} & 0 \\ 0 & 0 & 0 \end{bmatrix}$
19	TmPtIn	[0,0,1]	$[C_{2c}\|\|\mathcal{M}_c],\ [C_{2c}\|\|E],\ [C_{2d'}\|\|C_{2d}]\ d'/d = [1,\bar{1}/1,0]$	$\begin{bmatrix} 0 & 0 & 0 \\ 0 & 0 & 0 \\ \chi_{zx} & -\frac{1}{\sqrt{3}}\chi_{zx} & 0 \end{bmatrix}$
20	$YBaFe_4O_7$	n=[-1,0,-0.49]	$[C_{2n}\|\|E],\ [C_{2b}\|\|C_{2b}]$	$\begin{bmatrix} \chi_{xx} & 0 & \chi_{xz} \\ 0 & 0 & 0 \\ \frac{\sqrt{6}+\sqrt{2}}{4}\chi_{xx} & 0 & \frac{\sqrt{6}+\sqrt{2}}{4}\chi_{xz} \end{bmatrix}$
21	$TbMn_2O_5$	[0,0,1]	$[E\|\|\mathcal{M}_c],\ [C_{2c}\|\|E]$	$\begin{bmatrix} 0 & 0 & 0 \\ 0 & 0 & 0 \\ \chi_{zx} & \chi_{zy} & 0 \end{bmatrix}$
22	$La_{\frac{3}{8}}Ca_{\frac{5}{8}}MnO_3$	[0,1,0]	$[C_{2c}\|\|C_{2a}],\ [C_{2b}\|\|\mathcal{M}_b],\ [C_{2b}\|\|E]$	$\begin{bmatrix} 0 & 0 & 0 \\ 0 & 0 & \chi_{yz} \\ 0 & 0 & 0 \end{bmatrix}$
23	Er_2Pt	[0,1,0]	$[C_{2a}\|\|C_{2c}],\ [C_{2b}\|\|E],\ [E\|\|\mathcal{M}_b]$	$\begin{bmatrix} 0 & 0 & 0 \\ \chi_{yx} & 0 & 0 \\ 0 & 0 & 0 \end{bmatrix}$
24	$NaNdFeWO_6$	n=[0.01,0,1]	$[C_{2n}\|\|E]$	$\begin{bmatrix} 0 & 0 & 0 \\ 0 & 0 & 0 \\ \chi_{zx} & \chi_{zy} & \chi_{zz} \end{bmatrix}$
25	$BaNiTe_2O_7$	n =[1,0.26,0]	$[C_{2n}\|\|E],\ [C_{2c}\|\|C_{2c}]$	$\begin{bmatrix} \chi_{xx} & \chi_{xy} & 0 \\ 0.26\chi_{xx} & 0.26\chi_{xy} & 0 \\ 0 & 0 & 0 \end{bmatrix}$
26	$PrMn_2O_5$	[0,0,1]	$[E\|\|\mathcal{M}_c],\ [C_{2b}\|\|\mathcal{M}_a],\ [C_{2c}\|\|E]$	$\begin{bmatrix} 0 & 0 & 0 \\ 0 & 0 & 0 \\ \chi_{zx} & 0 & 0 \end{bmatrix}$
27	$DyFe_4Ge_2$	[0,0,1]	$[E\|\|\mathcal{M}_c],\ [C_{2b}\|\|\mathcal{M}_a],\ [C_{2c}\|\|E]$	$\begin{bmatrix} 0 & 0 & 0 \\ 0 & 0 & 0 \\ \chi_{zx} & 0 & 0 \end{bmatrix}$

No.	Name	Spin pol.	SPG generators	NREE tensor
28	$DyMn_2O_5$	[0,0,1]	$[E\|\|\mathcal{M}_c],\ [C_{2c}\|\|E],\ [C_{2a}\|\|\mathcal{M}_a]$	$\begin{bmatrix} 0 & 0 & 0 \\ 0 & 0 & 0 \\ \chi_{zx} & 0 & 0 \end{bmatrix}$
29	Li_2MnGeO_4	n=[1,0,-0.53]	$[C_{2b}\|\|\mathcal{M}_b],\ [C_{2n}\|\|E]$	$\begin{bmatrix} 0 & \chi_{xy} & 0 \\ 0 & 0 & 0 \\ 0 & -0.45\chi_{xy} & 0 \end{bmatrix}$
30	$Tm_5Ni_2In_4$	[0,0,1]	$[E\|\|\mathcal{M}_c],\ [C_{2c}\|\|E]$	$\begin{bmatrix} 0 & 0 & 0 \\ 0 & 0 & 0 \\ \chi_{zx} & \chi_{zy} & 0 \end{bmatrix}$
31	$DyBe_{13}$	[0,0,1]	$[E\|\|C_{2c}],\ [C_{2c}\|\|E]$	$\begin{bmatrix} 0 & 0 & 0 \\ 0 & 0 & 0 \\ 0 & 0 & \chi_{zz} \end{bmatrix}$
32	$YbLuCoMnO_6$	[0,1,0]	$[C_{2b}\|\|C_{2b}],\ [C_{2b}\|\|E]$	$\begin{bmatrix} 0 & 0 & 0 \\ 0 & \chi_{yy} & 0 \\ 0 & 0 & 0 \end{bmatrix}$
33	Yb_2CoMnO_6	[0,1,0]	$[C_{2b}\|\|C_{2b}],\ [C_{2b}\|\|E]$	$\begin{bmatrix} 0 & 0 & 0 \\ 0 & \chi_{yy} & 0 \\ 0 & 0 & 0 \end{bmatrix}$
34	$NiCr_2O_4$	[1,0,0]	$[C_{2a}\|\|E],\ [C_{2c}\|\|C_{2c}],\ [E\|\|C_{2a}]$	$\begin{bmatrix} \chi_{xx} & 0 & 0 \\ 0 & 0 & 0 \\ 0 & 0 & 0 \end{bmatrix}$
35	$CsCrF_4$	[0,0,1]	$[E\|\|\mathcal{M}_c],\ [C_{2b}\|\|C_{2b}],\ [C_{2c}\|\|E]$	$\begin{bmatrix} 0 & 0 & 0 \\ 0 & 0 & 0 \\ \chi_{zx} & \frac{1}{\sqrt{3}}\chi_{zx} & 0 \end{bmatrix}$
36	$CsCr_{0.98}Al_{0.02}F_4$	[0,0,1]	$[E\|\|\mathcal{M}_c],\ [C_{2c}\|\|E],\ [C_{2d}\|\|C_{2b}]$ d=[110]	$\begin{bmatrix} 0 & 0 & 0 \\ 0 & 0 & 0 \\ \chi_{zx} & 0 & 0 \end{bmatrix}$
37	Cu_2FeSiS_4	n=[-0.17,0,1]	$[C_{2d}\|\|\mathcal{M}_b],\ [C_{2n}\|\|E]$ d=$[1,0,0.17]$	$\begin{bmatrix} 0 & \chi_{xy} & 0 \\ 0 & 0 & 0 \\ 0 & \frac{-1}{0.17}\chi_{xy} & 0 \end{bmatrix}$
38	Cu_2MnGeS_4	n=[1,0,0.35]	$[C_{2d}\|\|\mathcal{M}_b],\ [C_{2n}\|\|E],$ d =[0.35,0,-1]	$\begin{bmatrix} 0 & \chi_{xy} & 0 \\ 0 & 0 & 0 \\ 0 & 0.35\chi_{xy} & 0 \end{bmatrix}$
39	Cu_2MnSiS_4	n =[1,0,0.86]	$[C_{2n}\|\|E],\ [C_{2b}\|\|\mathcal{M}_b]$	$\begin{bmatrix} 0 & \chi_{xy} & 0 \\ 0 & 0 & 0 \\ 0 & 0.86\chi_{xx} & 0 \end{bmatrix}$
40	$Ni_2Mo_3O_8$	[0,1,0]	$[C_{2b}\|\|E],\ [C_{2c}\|\|C_{2c}],\ [E\|\|\mathcal{M}_b]$	$\begin{bmatrix} \chi_{xx} & \frac{1}{\sqrt{3}}\chi_{xx} & 0 \\ \sqrt{3}\chi_{xx} & -\chi_{xx} & 0 \\ 0 & 0 & \chi_{xx} \end{bmatrix}$
41	NdSbTe	[0,1,0]	$[C_{2b}\|\|E],\ [E\|\|\mathcal{M}_b]$	$\begin{bmatrix} 0 & 0 & 0 \\ \chi_{yx} & 0 & \chi_{yz} \\ 0 & 0 & 0 \end{bmatrix}$

References

[1] Ashcroft, N. W. & Mermin, N. D. Solid State Physics. In *Solid State Phys.*, iii (Elsevier, Philadelphia, 2014), internat. edn. URL http://linkinghub.elsevier.com/retrieve/pii/B9780123850300000207. arXiv:1011.1669v3.

[2] Lu, D. & Liu, P. Rationalization of the Hubbard U parameter in CeOx from first principles: Unveiling the role of local structure in screening. *J. Chem. Phys.* **140** (2014).

[3] Luo, Y. *et al.* CeNiAsO: An antiferromagnetic dense Kondo lattice. *J. Phys. Condens. Matter* **23** (2011).

[4] Wu, S. *et al.* Incommensurate Magnetism Near Quantum Criticality in CeNiAsO. *Phys. Rev. Lett.* **122**, 197203 (2019). URL https://link.aps.org/doi/10.1103/PhysRevLett.122.197203. 1707.09645.

[5] Gallego, S. V. *et al.* MAGNDATA : towards a database of magnetic structures. II. The incommensurate case. *J. Appl. Cryst* **49**, 1941–1956 (2016). URL http://scripts.iucr.org/cgi-bin/paper?S1600576716015491http://dx.doi.org/10.1107/S16005767160154911941.
